# Glycyrrhizin and boswellic acids, the golden nutraceuticals: multitargeting for treatment of mild–moderate COVID-19 and prevention of post-COVID cognitive impairment

**DOI:** 10.1007/s10787-022-01062-3

**Published:** 2022-09-22

**Authors:** Adel A. Gomaa, Yasmin A. Abdel-Wadood, Mohamed A. Gomaa

**Affiliations:** 1 Department of Pharmacology, Faculty of Medicine, Assiut University, Assiut, Egypt; 2 Faculty of Agriculture, Assiut University, Assiut, Egypt; 3 Department of Plastic Surgery, Faculty Medicine, Assiut University, Assiut, Egypt

**Keywords:** Glycyrrhizin, Boswellic acids, COVID-19, Antiviral, Antiinflammatory, Clinical trials, Cognitive impairment

## Abstract

Breakthrough infections have been reported in fully vaccinated persons. Furthermore, rebound symptoms have been reported following the new FDA granted emergency use to combat SARS-CoV-2. Glycyrrhizin (GR) and boswellic acids (BAs) combination has been shown to have highly successful actions against COVID-19 in our recent clinical trial. However, the study is limited by the small sample size, and therefore, the aim of this article is to comprehensively evaluate recent evidence on the efficacy of GR and BAs in preventing the development of COVID-19 in patients with mild and moderate infections and in preventing post-COVID-19 cognitive impairment, which is the most important symptom after recovery from Covid-19 disease. We have reviewed and discussed information published since the outbreak of the COVID-19 pandemic until July 2022 on preclinical (in vivo, in vivo and bioinformatics) and clinical studies related to the antiviral, anti-inflammatory and immunomodulatory activity of Gr and BAs. Sixteen studies were performed to determine the efficacy of GR against SARS-CoV-2. Ten studies were used primarily for in vitro and in vivo assays and six used molecular docking studies. However, the antiviral activity of BAs against SARS-CoV-2 was determined in only five studies using molecular modeling and bioinformatics. All these studies confirmed that GR n and BAs have strong antiviral activity and can be used as a therapeutic agent for COVID-19 and as a protective agent against SARS-CoV-2. They may act by inhibiting the main protease SARS-CoV-2 (Mpro) responsible for replication and blocking spike protein-mediated cell entry. Only seven rigorously designed clinical trials regarding the usefulness of GR, BAs or their combinations in the treatment of COVID-19 have been published as of July 2022. Although there is no clinical study regarding the treatment of cognitive impairment after COVID-19 that has been published so far, several preclinical and clinical studies have demonstrated the potential effect of GR and BAs in the prevention and treatment of cognitive impairment by inhibiting the activity of several molecules that activate inflammatory signaling pathway. In conclusion, the findings of our study documented the beneficial use of GR and BAs to treat SARS-CoV-2 and its variants and prevent post-COVID cognitive impairment. However, it warrants further studies with a larger randomized sample size to ensure that the studies have sufficient evidence of benefits against COVID-19 and post-COVID-19 symptoms.

## Introduction

According to WHO, the COVID-19 pandemic continues to threaten public health systems worldwide (WHO 2022). Breakthrough infections have been reported in fully vaccinated persons (Liu et al. 2022). Furthermore, it is becoming increasingly clear that many patients who become infected with SARS-CoV-2 subsequently develop a number of neuropsychiatric and cognitive complications defined as long COVID or post-COVID-19 condition. The National Institute for Health and Care Excellence (NICE) defined post-COVID-19 as on-going symptoms beyond 4–12 weeks after COVID-19 (WHO 2021). These symptoms may persist for a long time after recovery, which negatively affects the quality of life of patients. Fatigue and cognitive impairment, along with other enduring neuropsychiatric (e.g., depression) comprise the most common post-acute sequelae of SARS-CoV-2 (Renaud-Charest et al. 2021).

According to various studies, these post-COVID-19 symptoms are observed in about a third of patients who have suffered from COVID-19 of varying severity (Taquet et al. 2021; Nalbandian et al. 2021; Huang et al. 2021; Baker et al. 2021; Crivelli et al. 2022). There is increasing evidence that cognitive impairment is widely observed among the consequences of COVID-19 disease, even in patients who have developed mild disease symptoms (Tavares-Júnior et al. 2022; Crivelli et al 2022; Ceban et al. 2022; Hadad et al. 2022). The proportion of individuals with cognitive impairment after COVID-19 has been inconsistent, but a prospective follow-up study reported that 48–56% of patients may have cognitive impairment after severe COVID-19 infection (Miskowiak et al. 2022). It is not clear what is definitely the risk factor for developing cognitive impairment after recovery from COVID-19 but several studies have revealed that comorbidities, age, SARS-CoV-2-induced neuroinflammation, systemic inflammation, brain hypoxia, prolonged stay in intensive care unit, peripheral organ impairment and genetic predisposition may be the mechanism underlying cognitive impairment after COVID-19 (Haage and De Jager 2022; Crivelli et al. 2022).

Since the beginning of the pandemic, there have been tremendous efforts to find effective outpatient treatments for COVID-19. The development of treatments has been shown to be important for preventing severe infection especially in individuals who have a high risk of progressing to severe disease. Two groups for the treatment of COVID-19 are currently recommended for people with mild or moderate symptoms of COVID-19 (Queensland Health, 2022). These groups include antivirals that target vital steps in viral replication (antiviral therapy**)** such as nirmatrelvir plus ritonavir which is a peptidomimetic that inhibits the 3C-like protease, rendering it unable to process polyprotein precursors, thus preventing viral replication. It is taken orally in combination with low-dose ritonavir to inhibit CYP3A-mediated metabolism of nirmatrelvir. The other group is neutralizing antibodies targeting M spike protein (anti-spike monoclonal antibodies) such as Sotrovimab which is a recombinant human immunoglobulin monoclonal antibody targeting the spike protein receptor binding domain of SARS-CoV-2 (Bernal et al. 2022; Gottlieb et al. 2021; Ng TI et al. 2022; Hammond et al. 2022). On December 22, 2021, FDA granted emergency use authorization for nirmatrelvir plus ritonavir for non-hospitalized symptomatic patients with SARS-CoV-2 to prevent progression to severe disease (FDA Update, 2022). However, rebound symptoms after a period of improvement have been reported. Furthermore, several lab studies have recently shown that SARS-CoV-2 can mutate in ways that make it less susceptible to the most oral antivirals authorized to treat SARS-CoV-2 in the USA. SARS-CoV-2 accumulated three mutations at positions 50, 166 and 167 in the amino acid chain that reduced virus susceptibility to nirmatrelvir by 20-fold (Wang et al. 2022;Rubin 2022; Robert 2022; Alshanqeeti and Bhargava 2022; HAN Archive 2022).

Nutraceuticals, including a variety of phytochemicals isolated from medicinal plants, dietary supplements, and functional foods, have long been used as an adjuvant treatment for many disease conditions, including viral infections. Several clinical studies are currently reporting the beneficial role of nutraceuticals in the treatment and/or prevention of COVID-19 (Subedi et al. 2021; Chavda et al. 2022). GR/licorice extract and BAs/Boswellia extract are nutraceuticals that have already been shown to possess anti-inflammatory, immunomodulatory, and antiviral activity against SARS-CoV-2 (Gomaa and Abdel- Wadood 2021; Gomaa et al. 2021). GR and BAs combination has been shown to have highly successful actions against COVID-19 in our recent clinical trial. We anticipated that the combination of the antiviral agent, GR, and the anti-inflammatory agent, immunomodulator, BAs, might have a more beneficial effect against COVID-19 (Gomaa et al. 2022). However, the study is limited by the small sample size; therefore, the aim of this article is to comprehensively evaluate recent evidence on the efficacy of GR and BAs in preventing the progression of COVID in patients with mild–moderate infection and in preventing post-COVID cognitive impairment, the widely observed among the consequences of COVID-19 disease.

## Methods

We have reviewed information published through July 2022. Searches were conducted to find relevant articles and studies relating to the use of GR/licorice extract, and BAs/Boswellia extract in COVID-19. These studies demonstrated the effects, use and safety of GR/licorice extract and BAs/Boswellia extract in preventing/treating COVID-19 and post-COVID cognitive impairment, in both clinical and preclinical studies (in vitro, in vivo and bioinformatics). The studies were collected by searching on online electronic databases (academic libraries such as PubMed, Scopus, Medline, Embase, Web of Science and the Egyptian Knowledge Bank). Articles were excluded if they were not available in English, in an indexed journal or in the international clinical trial registration platform regarding clinical studies (RCT). The opinion papers, retrieval of rapid reviews, guidelines documents, scope reviews, and panel reviews were not considered for our review. All articles were then approved and reviewed by two people (AG, YA) for relevance and included in this review.

## Results and discussion

### Antiviral activity of glycyrrhizin and boswellic acids against SARS-CoV-2 and mechanism of action

Determining the in vitro antiviral activity of potentially effective candidates is a necessary step for clinical studies, as extrapolation from in vitro to in vivo assumes that in vivo cellular drug accumulation is similar to that seen in in vitro experiments (Bocci et al. 2020). Since the 1979s, many studies reported that glycyrrhizin showed antiviral effect directly by inhibiting the replication of various DNA and RNA viruses including SARS-CoV and disrupting viral uptake into the host cells at low concentrations without cytotoxicity or indirectly by activating the immune function (Gomaa and Abdel- Wadood 2021; Huan et al. 2021; Ng et al. 2021). After the WHO officially declared a pandemic on March 11, 2020, several studies were conducted to determine the effectiveness of GR in treating COVID-19 using an in vitro assay and in silico structure-based virtual screening/docking on both human host targets and viral proteins. Since the beginning of this pandemic, 16 studies have been carried out to identify the efficacy of GR against SARS-CoV-2. Ten studies primarily used in vitro and in vivo assays. Six used molecular docking studies. All these studies confirmed that GR can be used as a therapeutic agent for COVID-19 and as a preventive agent against SARS-CoV-2 (Table 1).

**Table 1:** Antiviral activity of glycyrrhizin (glycyrrhizic acid) and boswellic acids against SARS-CoV-2 and mechanism of action in articles published through July 2022

Active ingredient	Method of research	Major finding	Mechanism of actions	References
Glycyrrhizic acid (GB-1)	Cell culture ACE2/SARS-CoV-2 spike inhibitor screening assay	Glycyrrhizic acid showed prophylaxis action against different variants of SARS-CoV-2 infection	Glycyrrhizic acid inhibits binding between ACE2 and RBD with different mutations	Tsai et al. (2022)
Glycyrrhizinic acid (Glyc) and its derivative (acylation with nicotinic acid)	Antiviral activity against three strains of SARS-CoV-2 was tested in vitro on Vero E6 cells and MTT assay	They have low toxicity and a broad spectrum of antiviral action against three strains of SARS-CoV-2 and HIV pseudovirus infection	Interfered with virus entry into the target cell	Fomenko et al. (2022)
The triterpenoids licorice-saponin A3 (A3) and glycyrrhetinic acid	Experimental in vitro experiments in vivo and autodocking	GA and A3 from licorice potently inhibit SARS-CoV-2 infection by affecting entry and replication of the virus	By targeting nsp7 and the spike protein RBD,	Yi et al. (2021)
Glycyrrhizin	In vitro, cellular perturbations in lung cells, macrophages cultured in conditioned media from lung cells	Glycyrrhizin inhibited SARS-CoV-2 replication in Vero E6 cells without exhibiting cytotoxicity at high doses. Glycyrrhizin mitigated viral proteins induced lung cell pyroptosis and activation of macrophages and resolving hyper-inflammatory processes	Glycyrrhizin prevents SARS-CoV-2 S1 and Orf3a induced high mobility group box 1 (HMGB1) release and inhibits viral replication	Gowda et al. (2021)***
Glycyrrhizic acid (GA) nanoparticles (GANPs)	In vitro and in vivo studies in mouse model of COVID-19	GANPs and GA exert antiviral and anti-inflammatory effects, relieving organ damage and conferring a significant survival advantage to infected mice	GANPs and GA could prominently suppress the proliferation of COVID-19	Zhao et al. (2021a, b)
Licorice extract and glycyrrhizin	Computational and in vitro experimental investigations	In vitro studies demonstrated robust anti-SARS-CoV-2 activity of licorice and glycyrrhizin under different treatment protocols (simulations treatment with viral infection, post-infection treatment, and pre-treatment,	Multiple mechanisms for action. Inhibitors for SARS-CoV-2 main protease (Mpro)	Tolah et al. (2021)
Glycyrrhizic acid	Experimental in vitro and Docking analysis	Glycyrrhizic acid inhibits SARS-CoV-2 infection	By blocking spike protein-mediated cell attachment. Molecules	Li et al. (2021)
Glycyrrhizic acid Comparing with ginsenoside Ra2, ginsenoside Rb3, berberine chloride	Combination of computer-aided drug design and in vitro biological verification	Glycyrrhizic acid was found to be the most efficient and nontoxic broad-spectrum anti-coronavirus molecule in vitro, especially, the significant effect on SARS-CoV-2	Glycyrrhizic acid performed the best in disrupting the interaction between the RBD of SARS-CoV-2 and ACE2	Yu et al. (2021)
*Glycyrrhiza glabra* extract contains 6.25% of glycyrrhizinic acid	Rats in normal or stress condition	This study provide evidence that glycyrrhiza glabra extract may reduce an entry point of SARS-CoV-2	*Glycyrrhiza glabra* root extract leads to a significant reduction in the expression of ACE2 in the small intestine	Jezova et al. (2021)
Glycyrrhizin	In vitro, Vero E6 cells	Glycyrrhizin potently blocks SARS-CoV-2 replication	Protease inhibitory activity of glycyrrhizin on the SARS-CoV-2 main protease Mpro	van de Sand et al (2021)
Ephedra and glycyrrhiza	Molecular docking	Ephedra and glycyrrhiza have antiviral activity against COVID-19 through multiple targets and pathways	The active compounds from the Ephedra-Glycyrrhiza pair bound well to COVID-19-related targets, including the main protease (Mpro, also called 3CLpro), the spike protein (S protein), and the angiotensin-converting enzyme 2 (ACE2)	Li et al. (2021)
Licorice and glycyrrhizic acids	Molecular docking, Autodock vina software	Glycyrrhizic acid could be considered as the best molecule from licorice, which could find useful activity against SARS-CoV-2	Glycyrrhizic acid was found to be best suited for the binding pocket of spike glycoprotein and inhibited the entry of the virus into the host cell	Sinha et al. (2021)
Glycyrrhizin	Blind docking approach, AutoDock	Glycyrrhizin is inhibitors against ACE2 host receptor binding of SARS-CoV-2	Glycyrrhizin show strong binding with ACE2. These compounds bind at the H1-H2 binding pocket	Ahmad et al. (2021)
Natural antioxidants including glycyrrhizin and its metabolite 18-glycyrrhetinic acid	Molecular docking, and MD simulations	Glycyrrhizin was found as the best ligand showing strong inhibition of five SARS-CoV-2 proteins	Glycyrrhizin and its metabolite 18-glycyrrhetinic acid have shown a strong binding affinity for MPro, helicase, RdRp, spike, and E-channel proteins, while a flavonoid Baicalin also strongly binds against PLpro and RdRp	Rehman et al. (2021)
56 licorice compounds	Silico interaction of main licorice components against SARS-CoV-2 infection	Glycyrrhizic acid, considered as a licorice major active ingredient, have a significant antiviral effects	Glycyrrhizic acid have the highest affinity to all targets	Maddah et al. (2021)
β-Boswellic acid and glycyrrhizic acid comparing with many compounds	Molecular docking studies to identify binding of medicinal metabolites with SARS-CoV-2 E protein	Out of screened compounds, β-boswellic acid (B. serrata) was found to be most suitable, along with glycyrrhizic acid (G. glabra) antiviral activities against enveloped viruses	They are strong SARS-CoV-2 E protein inhibitors	Fatima et al. (2022)
Bioactive compounds from *Boswellia serrata*	Computational method. AutoDock and PATCHDOCK	It was found that Euphane possesses most significant inhibitory potential against all of four receptors of virus	The top five ligands, bioactive compounds, bind to the catalytic dyad amino acid residues of Mpro by different bonding interactions	Roy and Menon (2022)
Acetyl‐11‐keto‐β‐boswellic acid (AKBA), 11‐keto‐β‐boswellic acid (KBA), and β‐boswellic acid (BBA)	Molecular modeling and bioinformatics	BAs have been reported to possess antiviral properties as antiviral drugs	The data of this bioinformatics study suggest that BAs target SARS‐CoV‐2 viruses on an atomic scale on three functional proteins, which in turn are responsible for human cell adhesion or viral RNA replication	Caliebe et al. (2021)
Alpha-boswellic acid (ABA) and beta-boswellic acid (BBA) which are active components	Docking studies	The binding of the ABA and BBA with the spike of the virus could inhibit its reproduction The LC50 values indicated that a high amount of ABA and BBA could be used safely in the human body	Binding energy indicates the high affinity between the spike proteins with the studied compounds	Kadhim et al. (2021)

The antiviral activity of glycyrrhizin and its derivatives has provided scientific evidence guiding clinical applications. All studies classified the mechanism of actions underlying the direct antiviral effect into two mechanisms: (1) inhibition of virus proteins (spike (S) proteins) mediating cell binding (ACE2) and blocking virus entry. (2) Inhibition of 3C-like protease or main protease (Mpro) and inhibits virus replication and aggregation in the host. Compared with other biologically active substances, in molecular docking with a biological test in vitro, it was found that GR, is the most effective and safe molecule to combat the coronavirus in general and specifically affect SARS-CoV-2. It was most effective in blocking the interaction between the domain-binding receptor (DBR) (more specifically, nsp7) of the spike protein of SARS-CoV-2 and ACE2 (Yu et al. 2021; Li et al.; Jezova et al. 2021; Yi et al. 2021).

In Vero E6 cells, GR fought SARS-CoV-2 by active inhibition of the main protease (Mpro) of SARS-CoV-2 and inhibited SARS-CoV-2 replication (van de Sand et al. 2021; Gowda et al.^ 2021^) In another study using computational and experimental investigations in vitro, licorice and GR treatment when used concurrently, post-infection, and pre-treatment have demonstrated potent anti-SARS-CoV-2 ability through two mechanisms of action as SARS-CoV-2 inhibitors for main protease (Mpro) responsible for replication and prevent cell binding mediated by spike protein. The results of this study suggested that GR can be used as a protective agent against COVID-19 (Tolah et al. 2021). In vivo study on a mouse model of COVID-19 combined with in vitro experiments; GR nanoparticles exert antiviral and anti-inflammatory effects, preventing organ damage and death by prominently suppressing the replication of COVID-19 (Zhao Z et al. 2021).

**Fig. 1:**
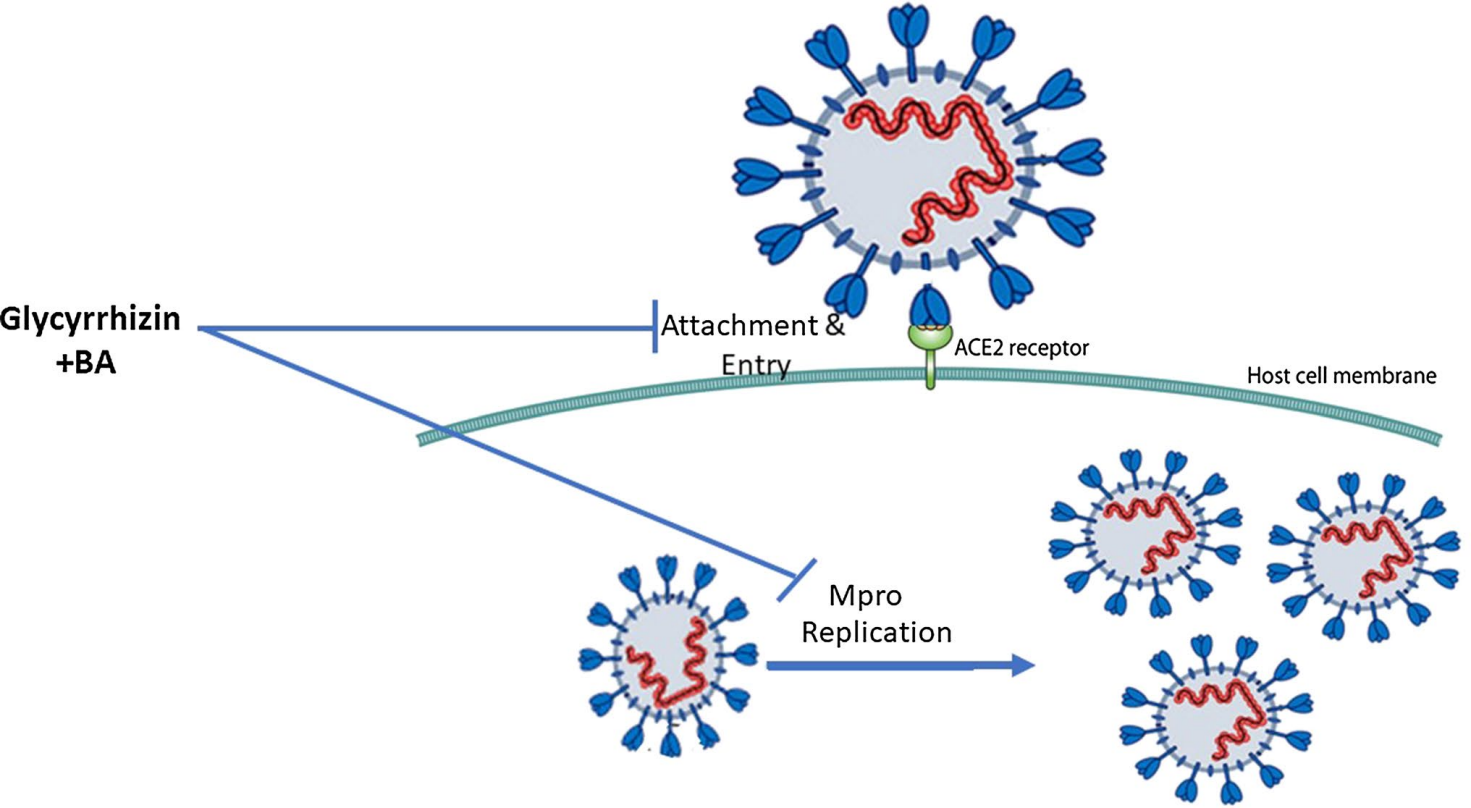
Mechanism of antiviral activity of glycyrrhizin and boswellic acids

Several studies have reviewed the antiviral activity of BAs/Boswellia extract in vitro, in vivo and theoretically (Gomaa et al. 2021; Jamshidi et al. 2022). The antiviral activity of BAs against SARS-CoV-2 was determined using molecular modeling and bioinformatics. No in vitro or in vivo studies have been published determining the efficacy of boswellic acids on SARS-CoV-2. To date, only four theoretical studies have been published using bioinformatics methods (Table 1). Recently, the activity of BAs, GR and several natural compounds was determined by studying molecular docking to measure the binding of these compounds to the SARS-CoV-2 E protein. BAs and GR were found to have strong antiviral activities against enveloped viruses because they are inhibitors of the SARS-CoV-2 E protein (Fatima et al. 2022). Data from other bioinformatics studies suggested that BAs possess antiviral properties because BAs target SARS-CoV-2 viruses at an atomic scale on three functional proteins, which are responsible for human cell adhesion (spikes of the virus) or viral RNA replication (main proteins of SARS-CoV-2, Mpro) (Caliebe et al. 2021; Kadhim et al. 2021; Roy and Menon 2022). However, experimental in vitro and in vivo studies are required to document the antiviral activities of BA/Boswellia extract against SARS-CoV-2 as demonstrated with GR. Meanwhile, the mechanism of action of GR and BA against SARS-CoV-2 is similar and can be improved using them together as with HCV combination therapy (Fig. 1).

### Effect of glycyrrhizin and boswellic acids on inflammatory pathways triggered by SARS-CoV-2 in vitro and in vivo animal models

It has been established that all complications of COVID-19 infection result from the hyperinflammation associated with SARS-CoV-2 infection. The SARS-CoV-2 N protein enhances NLRP3 inflammasome activation to induce excessive inflammation (Pan et al. 2021). This finding suggests that drugs with anti-inflammatory properties and NLRP3 inhibition may play an important role in the treatment of COVID-19. The anti-inflammatory activity of BAs and GR has been documented in in vitro, in vivo, and clinical studies. In these studies, experimental inflammation was induced by an inflammatory stimulus other than SARS-CoV-2. Recently, the immunomodulatory and anti-inflammatory properties of BAs and GR were reviewed by many investigators (Gomaa et al. 2021; Gomaa and Abdel-wadood 2021; Zheng et al. 2021; Renda et al. 2022). From the outbreak of the COVID-19 pandemic until July 2022, only six studies on the treatment of COVID-19 inflammation (in vivo, in vitro and bioinformatics) have been published. These studies confirm the anti-inflammatory activity of glycyrrhizin against inflammation caused by SARS-CoV-2 (Table 2).

**Table 2:** Effect of glycyrrhizin on inflammatory pathways triggered by SARS-CoV-2 in articles published through July 2022

Active ingredient	Method of research	Major finding	Mechanism of actions	References
Glycyrrhetinic acid (GA)	Computational, in vivo and in vitro experiments	This study provides theoretically and practically basis for GA to be used as a promising drug for treating COVID-19 cytokine storm	GA may exert anti-inflammatory effects through multiple targets and pathways. GA had good affinity with TNF, IL-6, MAPK3, PTGS2, PPARG and ESR1 and act by inhibiting their release	Li et al. (2022)
Glycyrrhizin	Mice sepsis induce—acute respiratory distress syndrome (ARDS) model	Glycyrrhizin alleviates sepsis-induced acute respiratory distress syndrome (ARDS)	Via suppressing of HMGB1/TLR9 pathways and neutrophils extracellular traps formation	Gu et al. (2022)
Glycyrrhizin	Cell culture, culture of macrophages in lung cell	Dual ability of glycyrrhizin to concomitantly block virus replication and inhibit proinflammatory mediators	Glycyrrhizin prevents SARS-CoV-2 (S1 and Orf3a) induced high mobility group box 1 (HMGB1) release which induce high release of proinflammatory cytokines IL-1β, IL-6 and IL-8, as well as ferritin from macrophages	Gowda et al. (2021)
Glycyrrhizic acid, nanoparticles glycyrrhizic acid	In vitro and in vivo investigations. Mouse model of COVID-19 and animal model of excessive inflammation	They exert antiviral and anti-inflammatory effects, relieving organ damage and conferring a significant survival advantage to infected mice	They reduce proinflammatory cytokine production caused by MHV-A59 or the N protein of SARS-CoV-2	Zhao et al. (2021a, b)
Glycyrrhizic acid (GA)	Computational network pharmacology and bioinformatic analysis	GA may be a suitable molecular drug for ameliorating excessive inflammation triggered by SARS-CoV-2	Through inhibition of the IL-17, IL-6, and TNF-α signaling pathways	Zheng et al. (2021)
Glycyrrhizic acid in combination with vitamin c and curcumin	Computational system biology tools measuring biological processes and pathways have been well documented in CoV infection studies	Glycyrrhizic acid in combination with vitamin c and curcumin may be helpful in regulating immune response to combat CoV infections and inhibit excessive inflammatory responses to prevent the onset of cytokine storm	Regulate innate immune response by acting on NOD-like and Toll-like signaling pathways to promote interferons production, activate and balance T cells, and regulate the inflammatory response by inhibiting PI3K/AKT, NF-κB and MAPK signaling pathway	Chen et al. (2021)

Interestingly, one of these studies provides a theoretical and practical basis for using glycyrrhizin as a promising drug to treat the COVID-19 cytokine storm that is a major complication of COVID-19 disease (Li H et al. 2022). Furthermore, in mice sepsis ARDS model of acute respiratory distress syndrome which is the most important complications of COVID-19, GR prevents the development of acute respiratory distress syndrome (Gu et al. 2022). GR exerts this effect through multiple mechanism and pathways (Li H et al. 2022). One of these mechanism depend on prevention of SARS-CoV-2 (S1 and Orf3a protein) induced HMGB1 release which induce high release of proinflammatory cytokines IL-1β, IL-8, and IL-6 (Gu et al. 2022; Gowda et al. 2021). Another suggestion involves inhibition of NLRP3 inflammasome inflammatory pathway that is activated by SARS-CoV-2 infection and causes excessive inflammation (Zhao et al. (2021a, b); Wang et al. 2022). Recently, Gu et al (2022) suggested that GR inhibits HMGB1/TLR9 pathways and neutrophils extracellular traps formation. In addition, Zheng et al. (2021) suggested that GA inhibit the inflammation by inhibiting the key targeting to COVID-19 that activates the response to reactive oxygen species.

Several studies in vivo and in vitro confirmed the immunomodulatory and anti-inflammatory activity of BAs/Boswellia extract (Khajehdeh et al. 2022); however, no study demonstrated the anti-inflammatory or immunomodulatory activity of BA in animal model of COVID-19 yet. Recently, Aldahlawi et al. (2020) and Zimmermann-Klemd et al. (2020) demonstrated that *Boswellia sacra* essential oil, Boswellia extract and BAs exert immunomodulatory effects on T cells and dendritic cells where it deviates the differentiation of monocytes into immature DCs. More recently, according to in vitro LPS-induced inflammation on H9C2 cells, an in vivo zebrafish larval model and molecular docking study, Boswellia extract and BAs inhibited inflammation and cytotoxicity and showed strong redox activity. The mechanism of action may be by increasing anti-inflammatory activity by decreasing specific inflammatory gene expression (iNOS, TNF-α, IL-1, and COX-2) (Siddhu et al. 2022; Taherzadeh et al. 2022). Taken together, the results of the above studies show evidence that GA and BA suppress SARS-CoV-2-induced inflammation through inhibition of the NLRP3 inflammasome pathway and HMGB1 release. Moreover, its immunomodulatory activity contributes to preventing the development of cytokine storms.

### Efficacy of glycyrrhizin/licorice extract and boswellic acids/Boswellia extract in the treatment of COVID-19 in clinical trials

GR/licorice was used in clinical trials to treat liver disease, viral infections (HCV, HBV), gastrointestinal disorders, oral diseases, and various skin disorders (Kwon et al. 2020; Huan et al. 2021; Leite et al. 2022) while BAs/Boswellia extract was used to treat osteoarthritis, ischemic stroke, cognitive impairment post-traumatic brain injury, MS, bronchial asthma and brain tumors (Baram et al. 2019; Abdel-Tawab et al. 2021; Varma et al. 2021). The pharmacological bases for the use of GR/licorice and BAs/Boswellia extract in clinical trials have been attributed to their anti-inflammatory, antioxidant and immunomodulatory properties. Since the outbreak of COVID-19, there have been few clinical studies on GR/licorice or BAs/Boswellia extract as a potential treatment for COVID-19, and there are many studies on Chinese prescriptions with licorice as a major ingredient (Sun et al. 2021). Only seven clinical trials with a rigorous design regarding the usefulness of GR, BAs and their combinations have been published through July 2022. These studies provide evidence of the benefit of using GR and BAs in COVID-19 management (Table 3).

**Table 3:** Effect of glycyrrhizin and boswellic acids on COVID-19 disease in clinical trials published through July 2022

Intervention/drugs	Patients	Design	Trial ID	Main outcomes	References
Glycyrrhizin capsule (GR) (60 mg) and boswellic acids (BA) capsule (200 mg) twice daily for 14 days	50 hospitalized patients with moderate COVID‑19 infection	Randomized, double-blind, placebo-controlled trial single-center trial	NCT04487964	GR + BA combination was effective in preventing mortality, shortening the time to recovery and improving prognosis or decreasing clinical status score on a 7-point scale. The laboratory parameters show significant difference between serum CRP and % lymphocyte of placebo group and intervention group supporting the improvement by GR + BA. It is safe, inexpensive, combination may be considered for use in mild to moderate infections of SARS-CoV-2 or COVID-19 variants	Gomaa et al. (2022)
Diammonium glycyrrhizinate (DG) in dose of 150 mg + vitamin C (VC)	207 COVID-19 patients from Tongji Hospital at Huazhong University (Wuhan, China)	Retrospective, single-center, observational study	Institutional Review Board of Ruijin Hospital, Shanghai Jiao Tong University School of Medicine (No. (2020) Linlun-34th)	DG + VC could reduce the incidence of new-onset complications in COVID-19 patients, and might influence the immune response in these patients. This results suggested that combined treatment of DG + VC is promising candidate for preventing the deterioration of COVID-19 patients	Tan et al. (2022)
Intranasal and oropharyngeal delivery of povidone-iodine 0.5% and glycyrrhizic acid 2.5 mg/ml	200 patients	Randomized-Blinded Controlled Study	PACTR registry under the number PACTR202101875903773	Combined PVI-GA nasal and oropharyngeal spray accelerates both laboratory and clinical recovery of SARS-CoV-2-infected patients in the early phases of the disease and reduces the household spread of the virus	Elsersy et al. (2022)
Viusid is natural product containing glycyrrhizinic acid 30 ml every 8 h for 21 days	60 hospitalized patients with mild to moderate symptoms of COVID-19	Randomized, open-label, controlled trial	MBAL “Sveti Mina” (RCT001/P4/2020)	Its use lead to faster recovery of the patients, decreasing of the hospital stay and milder course of the disease, with good safety and tolerability	Petrov et al. (2021)
Aftogel (licorice extract) oral mucoadhesive patches	125 outpatient with positive real-time PCR	Triple-blind randomized clinical trial	RCT20181208041886N2	The patch is effective in the eradication of SARS-CoV-2, which has colonized the nasopharyngeal area. Hence, this drug product has the potential for evaluation as a prophylactic	Pourahmad et al. (2022)
Inflawell Syrup (Boswellia extract formulation enriched for boswellic acids (BAs), 10 ml of syrup thrice daily (400 mg BAs)	47 hospitalized patients with moderate COVID-19	Randomized placebo-controlled double-blind clinical trial	IRCT20170315033086N10. IRCT is a primary registry in the WHO registry network	The treatment with BAs resulted in shorter hospital stay, alleviation of COVID-19 clinical symptoms, a significant decrease in the percentage of neutrophils and neutrophil-to-lymphocyte ratio (NLR) and decline in the level of proinflammatory cytokines	Barzin Tond et al. (2022)
Essential oil blend, the main active ingredient is frankincense (*Boswellia carterii*) inhaled twice daily for 14 consecutive days	Forty women who continue to experience fatigue more than 5 months after acute COVID-19 infection	A randomized double-blind, placebo-controlled trial	NCT04980573	Individuals who inhaled the essential oil blend for 2 weeks had significantly lower fatigue scores after controlling for baseline scores. This intervention improve energy levels and mental fatigue, as well as vigor significantly	Hawkins et al. (2022)

Importantly, our study showed that the combinations of GR and BAs were highly successful against COVID-19 (Gomaa et al. 2022). In this study, patients with moderate SARS-CoV-2 received a GR capsule (licorice extract 300 mg contains 60 mg of GR) twice daily 1 h before the BAs capsule (Boswellia extract 300 mg contains 200 mg of BAs). BAs capsules were taken after meals. The treatment was continued for 14 days even if symptoms of infection disappeared before the end of the treatment course. The results of our trial showed that there was significant decrease in percentage of mortality in group that received GR + BAs compared to placebo group with significantly shorter recovery time, in the intervention group. Clinical status on the ordinal score scale showed a significant decrease in the score of the GR + BA group. Clinical deterioration occurred in 0 patients in the intervention group and in 5 patients in the placebo group who required admission to the ICU for mechanical ventilation. There was a significant decrease in CRP and an increase in the percentage of lymphocytes in the intervention group compared with the placebo group.

Interestingly, in our preliminary, uncontrolled trial (20 patients), no patients had long-COVID-19 or post-COVID-19 symptoms as cognitive impairment, but five patients experienced fatigue and one had tachycardia after treatment had ended. These patients continued to receive half the dose of treatment of licorice extract and *Boswellia serrata* gum with vitamin C and zinc for a further 14 days or until these symptoms disappeared. All symptoms disappeared a month after starting treatment (unpublished data). However, hospitalized patients attending our randomized clinical trial were not followed up after discharge from hospital.

In another randomized, double-blind, placebo-controlled clinical trial conducted in a rigorous design, evidence of the efficacy of BAs in improving clinical symptoms and inflammatory markers in patients with moderate COVID-19 was shown. BAs formulated as syrup have been used three times daily for 14 days. The average length of hospital stay or recovery time was significantly shorter in the BAs group than in the placebo group. Furthermore, clinical manifestations were significantly improved in the BA group more than in the placebo group. This study showed a significant decrease in inflammatory markers as the percentage of neutrophils, neutrophil-to-lymphocyte ratio (NLR), CRP, TNFα and IL-6 levels in the BAs group compared with the placebo (Barzin Tond et al. 2022).

Two other trials suggested that topical use of GR as a nasal or oropharyngeal spray or mucous patch was effective in eradicating SARS-CoV-2, which colonized the nasopharyngeal area and accelerated laboratory and clinical recovery of patients infected with SARS-CoV-2 in the early stages of the disease. Therefore, it has the potential to prevent the progress of the disease and be a prophylactic agent against COVID-19. It may reduce the spread of the virus and, therefore, may play an important role in controlling the outbreak of SARS-CoV-2 (Elsersy et al. 2022; Pourahmad et al. 2022). Furthermore, in a randomized, double-blind, placebo-controlled trial, 2 weeks of inhalation of frankincense (*Boswellia carterii*) with another blend of essential oil extracts improved energy levels in healthy COVID-19 survivors suffering from a lack of energy for more than 5 months after recovery (long COVID-19). In addition, the results of this study showed that this combination could significantly relieve fatigue among women experiencing fatigue after recovery from COVID-19 (Hawkins et al. 2022).

In retrospective, single-center, observational study, diammonium glycyrrhizinate (DG) combined with vitamin C significantly attenuated the prognoses of COVID-19 disease in 207 hospitalized patients. New onset of complications such as ARDS, acute liver injury and acute myocardial injury was significantly fewer in group of patients treated by diammonium glycyrrhizinate with vitamin C compared with the non-DG group (Tan et al. 2022). This study is further support for the use of glycyrrhizin/licorice extract in the treatment of COVID-19. However, one of the weaknesses of this study is the lack of registration of this study in an international clinical trial site. It is clear from previous results that GR/licorice extract may be more effective than BAs/Boswellia extract in fighting COVID-19 while the combination of GR and BAs was the best in combating COVID-19.

### Potential effect of glycyrrhizin/licorice extract and boswellic acids/Boswellia extracts in prevention of post-COVID-19 cognitive impairment

A recent study indicated that hospitalization and the severity of COVID-19 can increase the risk of post-COVID-19 AD **(**Li C et al. 2022**)**. However, another study showed that cognitive impairment was not predicted by age, pre-existing conditions, or severity of COVID-19 disease (Hadad et al. 2022). Whatever the potential mechanisms underlying the cognitive impairment after recovery from COVID-19, there is not yet a study that tests a specific drug to prevent or treat this symptom. Meanwhile, GR/licorice extract and BAs/Boswellia extract have been shown to treat and prevent the development of cognitive impairment resulting from many causes in experimental and clinical studies (Ravanfar et al. 2019; Rajabian et al. 2020; Gomaa et al. 2021; Haghaei et al. 2021; Siddiqui et al. 2021, Gong et al. 2022). Our first study on the effect of *Boswellia serrata* extract on cognitive impairment showed that *Boswellia serrata* extract was effective in the prevention and treatment of cognitive impairment in animal models by inhibiting oxidative stress and proinflammatory cytokines (Gomaa et al. 2019).

Based on evidences, it is concluded that inflammation and oxidative stress plays a crucial role in cognitive impairment pathophysiology. Systemic inflammation may play an important role in promoting neurodegeneration, and cognitive decline. Inflammation is characterized by increased blood levels of proinflammatory cytokines. Furthermore, increased levels of oxidative stress in the brain over cellular antioxidant defenses can damage cellular structures and increase accumulation of beta-amyloid and neurofibrillary tangles. Increased oxidative stress and systemic inflammation may occur as a result of events such as infection, chronic disease, physical and psychological stress, and cellular aging (Walker et al. 2019; Buccellato et al. 2021).

Interestingly, it has been observed that HMGB1 (High Mobility Group Box-1) is elevated largely in serum of COVID-19 patients. HMGB1 is a proinflammatory cytokine with a strong ability to stimulate the inflammatory response and its elevated level have been associated with disease severity and the development of a cytokine storm (Al‑kuraishy et al. 2022). Moreover, HMGB1 has been suggested to be elevated in neuroinflammation, cognitive impairment, postoperative cognitive dysfunction and cognitive impairment in the late stage of TBI (traumatic brain injury) (Paudel et al. 2020; Lin et al. 2021; Tan et al. 2021). GR has been confirmed to have antagonistic properties to HMGB1 and NLRP3 inflammasome which are activated by TLR2, the main contributor to neuroinflammation and cognitive dysfunction (Lin et al. 2021; Tan et al. 2021).

In addition, GR has been shown to have a blocking effect against lipopolysaccharide-induced neuroinflammation and cognitive impairment in C57 mice by suppressing activation of the TLR4 signaling pathway that inhibits production of proinflammatory cytokines in the brain of C57 mice (Cho et al. 2018; Liu et al. 2019). Furthermore, in aged mice, GR improves learning and memory in aged mice by modulating T/B cell proliferation through the inhibition of several genes related to macrophages and neutrophils (Jiang et al. 2020). There are few clinical studies that have shown the effectiveness of GR in managing cognitive dysfunction. In a randomized, placebo-controlled clinical study, elderly people with mild cognitive impairment (MCI) used licorice extract for 12 weeks with or without training, and it can slow or halt the progression of MCI by significantly reducing inflammatory markers (IL-1β and TNF-α) but their decrease was more pronounced in group used licorice with training (Kohanpour et al. 2017).

Several in vivo studies demonstrated the benefit of BAs/Boswellia extract in cognitive impairment and plasticity impairments induced in the animal model by LPS-induced excessive inflammation. It works by inhibiting inflammation and oxidative stress. It may modulate the activity of multiple molecular targets that influence the signaling pathways that contribute to the pathogenesis of cognitive impairment (Borooni et al. 2020; Marefati et al. 2020, 2021, 2022). Mohammed et al. (2022) observed that BA modulated the expression of parameters related to the Wnt/-catenin pathway and decreased the expression of TNF-α IL-1β, attenuated lipid peroxidation, and raised total brain antioxidants. Another study indicated that a possible mechanism for the beneficial effects of BAs may include inhibition of the 5-LOX/COX pathway in arachidonic acid metabolism, activation of Nrf2 (has anti-inflammatory activity), through binding to ARE (antioxidant response element) and inhibition of NF-kB (Siddiqui et al. 2021). Furthermore, BAs eliminate memory impairment by enhancing the activity of PPARγ and its downstream regulators, matrix metalloproteinase 2 genes in the hippocampus (Gunasekaran et al. 2021). In a double-blind, randomized, placebo-controlled clinical trial, the effect of BAs on cognitive impairment after traumatic brain injury (TBI) was investigated. The results of this study after 3 months of follow-up showed that BA was safe, well tolerated, and had a positive effect on the cognitive function of patients with TBI (Meshkat, 2022**)**. In another clinical trial, *Boswellia* *papyrifera* significantly improved visuospatial memory, but verbal memory was not changed (Sedighi et al. 2014).

Aforementioned data provide evidence that systemic inflammation associated with COVID-19 infection is a prominent cause of neurodegeneration, and cognitive impairment. Interestingly, not only preclinical studies but also studies conducted in clinical settings have well established the potential effect of GR/licorice and BAs/Boswellia extract in the prevention and treatment of cognitive impairment by inhibiting the activity of multiple molecules that activate the signaling pathways of inflammation.

## Limitations

This review has some limitations. The most obvious limitation is the small and few clinical trials. The absence of preclinical in vitro and in vivo studies testing the antiviral activity of BA is another limitation. Third, the lack of studies comparing the activity of GR alone or GR + BA with the newly approved anti-COVID-19. Fourth: there is no follow-up for patients after their discharge from the hospital. Fifth, the potential interaction between GR or BAs and standard protocol drugs has not been studied. Finally, all clinical studies were performed only on adult patients and the data may not be applicable to pediatric groups.

## Conclusions

Several studies demonstrated the antiviral, anti-inflammatory and immunomodulatory activity of GR and BAs. All these studies confirmed that GR or GR + BAs have strong antiviral activity and can be used as a therapeutic agent for COVID-19 and as a protective agent against SARS-CoV-2. Our recent clinical trial showed that the combination of GR and BAs was highly successful against COVID-19. There are several clinical and preclinical studies indicating the benefit of GR and BAs in preventing cognitive impairment due to systemic inflammation, such as in COVID-19. However, more randomized controlled trials with effective, large populations are needed to show a definitive conclusion about therapeutic efficacy of GR and BAs in treatment of COVID-19 and prevention of post-COVID-19 symptoms. Based on the safety, clinical trials, and preclinical evidence presented in this review together with the failure of newly approved antiviral agents, GR or GR with BAs, should be disseminated globally and systematically in the treatment of SARS-CoV-2 and its variants and prevention of post-COVID cognitive impairment. However, more randomized controlled trials with effective, large populations are needed to show a definitive conclusion about therapeutic efficacy of GR and BAs in treatment of COVID-19 and prevention of post-COVID-19 symptoms.

## Data Availability

Data on relevant human studies in the current review. https://drive.google.com/file/d/17YZvAhCFoBMV6vdnjLFzfNN52vtpCKri/view?usp=sharing.

## Abbreviations

ARDS: Acute respiratory distress Syndrome
*B. serrata*: *Boswellia serrate*
Bas: Boswellic acids
KBA: 11-Keto-*β*-boswellic acid
AKBA: 3-O-acetyl-11-keto-β- boswellic acid
ACE2: Angiotensin-converting enzyme 2
Bax: Bcl-2-associated X protein
COX-2: Cyclooxygenase-2
COVID-19: Coronavirus disease 2019
CRP: C-reactive protein
GR: Glycyrrhizin
HMGB1: High-mobility group box 1 protein
iNOS: Inducible nitric oxide synthase
IL-1β: Interleukin one beta
IL-6: Interleukin-6
JAK: Janus kinase
JNK: C-Jun N-terminal kinase
5-LOX: 5-Lipoxygenase
LTB4: Leukotriene B4
MERS-CoV: Middle East respiratory syndrome coronavirus
NLRP3: Nod-like receptor protein3
NF-κB: Nuclear factor kappa B
SARS-CoV: Severe acute respiratory syndrome coronavirus
TLR: Toll-Like Receptor 2
TNF-α: Tumor necrosis factor alpha
